# Examination of the Top Three Traumatic Experiences Among United States Service Members and Veterans with Combat-Related Posttraumatic Stress Disorder

**DOI:** 10.3390/bs15091211

**Published:** 2025-09-05

**Authors:** Kiara H. Buccellato, Casey L. Straud, Tabatha H. Blount, Wyatt R. Evans, Jennifer M. Hein, Elizabeth Santos, Willie J. Hale, Edna B. Foa, Lily A. Brown, Carmen P. McLean, Richard P. Schobitz, Bryann B. DeBeer, Joseph Mignogna, Brooke A. Fina, Brittany N. Hall-Clark, Christian C. Schrader, Jeffrey S. Yarvis, Vanessa M. Jacoby, Jose M. Lara-Ruiz, Kelsi M. Gerwell, Brett T. Litz, Eric C. Meyer, Barbara L. Niles, Stacey Young-McCaughan, Terence M. Keane, Alan L. Peterson

**Affiliations:** 1 Department of Psychiatry and Behavioral Sciences, University of Texas Health Science Center at San Antonio, 7703 Floyd Curl Drive, MC 7747, San Antonio, TX 78229, USA; casey.straud@utsa.edu (C.L.S.); blountt@uthscsa.edu (T.H.B.); wyattrevans@gmail.com (W.R.E.); santose1@uthscsa.edu (E.S.); willie.hale@utsa.edu (W.J.H.); fina@uthscsa.edu (B.A.F.); hallclark@uthscsa.edu (B.N.H.-C.); jacobyv@uthscsa.edu (V.M.J.); jose.m.lara-ruiz.civ@health.mil (J.M.L.-R.); gerwell@uthscsa.edu (K.M.G.); youngs1@uthscsa.edu (S.Y.-M.); petersona3@uthscsa.edu (A.L.P.); 2 Department of Psychology, University of Texas at San Antonio, 1 UTSA Circle, San Antonio, TX 78249, USA; 3 Rocky Mountain MIRECC for Suicide Prevention, U.S. Department of Veterans Affairs, 1700 N Wheeling Street, Building A2, Aurora, CO 80045, USA; bryann.debeer@va.gov (B.B.D.); joseph.mignogna@va.gov (J.M.); 4 Research and Development Service, South Texas Veterans Health Care System, 7400 Merton Minter, San Antonio, TX 78229, USA; 5 VA North Texas Health Care System, 4500 S Lancaster Road, Dallas, TX 75216, USA; 6 Department of Behavioral Health, Carl R. Darnall Army Medical Center, 6065 Santa Fe Avenue, Fort Hood, TX 76544, USA; jennifer.m.hein6.mil@health.mil (J.M.H.); christian.c.schrader.mil@health.mil (C.C.S.); jyarvis@tulane.edu (J.S.Y.); 7 Department of Psychology, St. Mary’s University, 1 Camino Santa Maria, San Antonio, TX 78228, USA; 8 Center for the Treatment and Study of Anxiety, Department of Psychiatry, University of Pennsylvania, 3535 Market Street, Suite 601N, Philadelphia, PA 19104, USA; foa@pennmedicine.upenn.edu (E.B.F.); lilybr@pennmedicine.upenn.edu (L.A.B.); 9 Dissemination and Training Division, National Center for PTSD, VA Palo Alto Health Care System, 795 Willow Road, Menlo Park, CA 94025, USA; carmen.mclean4@va.gov; 10 Department of Psychiatry and Behavioral Sciences, Stanford University, 291 Campus Drive, Stanford, CA 94305, USA; 11 Department of Behavioral Health, Brooke Army Medical Center, 3551 Roger Brooke Drive, Joint Base San Antonio-Fort Sam Houston, San Antonio, TX 78234, USA; richard.schobitz@hhs.gov; 12 U.S. Public Health Service Commissioned Corps Headquarters, 1101 Wootton Pkwy, Rockville, MD 20852, USA; 13 Anschutz Medical Campus, University of Colorado, 12631 E 17th Avenue, Aurora, CO 80045, USA; 14 School of Social Work, Tulane University, 127 Elk Place, New Orleans, LA 70112, USA; 15 Massachusetts Veterans Epidemiology Research and Information Center, VA Boston Healthcare System, 150 S Huntington Avenue, Boston, MA 02130, USA; litzb@bu.edu; 16 Department of Psychiatry, Chobanian and Avedisian School of Medicine, Boston University, 801 Massachusetts Avenue, 1st Floor, Boston, MA 02118, USA; barbara.niles@va.gov (B.L.N.); terry.keane@va.gov (T.M.K.); 17 Department of Counseling and Behavioral Health, University of Pittsburgh, 119 University Pl, Pittsburgh, PA 15213, USA; ecm77@pitt.edu; 18 Behavioral Science Division, National Center for PTSD at VA Boston Healthcare System, 150 South Huntington Avenue, Boston, MA 02130, USA

**Keywords:** posttraumatic stress disorder, military PTSD, trauma-focused treatment augmentation

## Abstract

Many trauma-focused psychotherapies for posttraumatic stress disorder (PTSD) focus on the most distressing trauma. However, military personnel are often exposed to multiple traumatic experiences. This study aimed to evaluate and categorize the top three traumatic experiences identified by United States (U.S.) military service members seeking treatment for PTSD and compare frequency of trauma types by demographic/military characteristics. Active duty service members and veterans (*N* = 110) with PTSD identified and ranked their top three most distressing experiences. Behavioral health professionals classified experiences according to one categorical and four dichotomous classification schemes. The categorical scheme included life threat to self, life threat to others, aftermath of violence, traumatic loss, moral injury by self, and moral injury by others. The Life Threat to Self classification represented the largest portion of categorical experiences (43%). Most experiences were dichotomously classified as military-related (86%), combat-related (70%), non-sexual (91%), and trainability (versus futility; 71%). Women were more likely to report sexual traumatic experiences and less likely to report military- and combat-related experiences. Military occupational specialty, number of deployments, time in military, active duty status, and marital status were also associated with different classification rates. There was noteworthy variability in types of experience across top three traumas, especially among certain subpopulations.

## 1. Introduction

Posttraumatic stress disorder (PTSD) is a pervasive issue within the United States (U.S.) military population who completed combat deployment after the 11 September 2001 attacks on America. Between 2002 and 2016, the incidence of PTSD increased from 1.24 to 12.94 per 1000 service members (Judkins et al., 2020). Dedert et al. (2009) found that 94% of U.S. military veterans reported exposure to at least one traumatic event, with 57% reporting exposure to three or more traumatic experiences. Jakob et al. (2017) found that 76% of veteran respondents reported exposure to four or more lifetime traumatic experiences.

PTSD is a mental health disorder characterized by behavioral, affective, and cognitive reactions to a potentially traumatic event (American Psychiatric Association, 2022). To meet clinician-evaluated diagnostic criteria for PTSD, individuals must have been exposed to an event that entailed “actual or threatened death, serious injury, or sexual violence”, must currently be experiencing symptoms from four defined symptom categories that have persisted for at least the past month, and must report that these symptoms have caused them distress or impairment to social or occupational functioning (American Psychiatric Association, 2022). Functional impairment is often seen in PTSD across multiple areas of daily life, including self-care, mobility, interpersonal relationships, community engagement, etc. (Jellestad et al., 2021). People may experience various posttraumatic stress symptoms after exposure to a potentially traumatic event, without meeting all requirements for a PTSD diagnosis. According to an epidemiological study of PTSD rates in a cross-national sample, only approximately 5.6% of trauma exposed individuals are diagnosed with PTSD in their lifetime (Koenen et al., 2017). Recovery trajectories can vary greatly among individuals with PTSD, with a meta-analysis evaluating long-term PTSD outcomes reporting remission rates of 6–92% across 25 studies (Steinert et al., 2015).

Many trauma-focused psychotherapies for PTSD prioritize a single most distressing trauma for treatment activities, referred to as the Criterion A event in the fifth edition of the *Diagnostic and Statistical Manual for Mental Disorders* (*DSM-5-TR*; American Psychiatric Association, 2022). Given the literature demonstrating that many service members are exposed to multiple traumatic experiences during military deployments, focusing on a single trauma in treatment may have drawbacks for some patients. Focusing on a single trauma may run the risk of failing to validate the importance of the other traumatic experiences. Additionally, while generalization of treatment benefits when focusing on the worst event can and does occur in many cases, this may not occur in all cases, particularly when different traumatic experiences elicit different symptoms (Guina et al., 2018).

The purpose of this study was to categorize and evaluate patterns of traumatic experiences reported by active duty service members and veterans with PTSD who had reported and ranked their top three most traumatic experiences from most to least distressing experience. Evaluation of the diversity of traumatic experiences reported by service members and veterans overall and across demographic and military-related subgroups when given the opportunity to address top three most distressing experiences could help inform the theoretical justification for future augmentation of trauma-focused treatments to address more than the single most distressing traumatic experience.

### Objectives

The first objective of this study was to evaluate and categorize the top three traumatic experiences identified by active duty service members and veterans seeking treatment for PTSD according to five trauma classification schemes, including one categorical and four dichotomous ratings. The second objective was to compare the frequency of traumatic experiences in each category to determine if significant patterns existed when comparing trauma types by demographic and military characteristics.

## 2. Materials and Methods

This study was a secondary analysis using data collected as part of a larger randomized clinical trial examining massed versus intensive outpatient (IOP) Prolonged Exposure therapy (PE; Peterson et al., 2018; Peterson et al., 2023). U.S. active duty military personnel and veterans who had deployed in support of a post-9/11 combat operation, experienced at least one deployment-related Criterion A event and met diagnostic criteria for PTSD were recruited through self- and provider-referral from military treatment facilities and Veteran’s Affairs facilities in Texas, USA. In the IOP-PE arm, participants addressed their top three traumatic experiences during imaginal exposure, starting with the one they reported as least (i.e., third most) distressing and working up to the most distressing event. Participants in the massed PE arm identified and addressed only their single most traumatic experience. Given this article’s objective of evaluating patterns of top three reported traumatic experiences, participants in the massed PE arm were excluded from these analyses. The top three traumatic experiences were identified and ranked using the Selection of Index Event for the Clinician-Administered PTSD Scale for DSM–5 (CAPS-5; Weathers et al., 2018). This process involved study therapists working with participants to confirm their experiences met DSM-5 Criterion A (i.e., involved direct exposure to or witnessing of an event involving death, threatened death, actual or threatened serious injury or sexual violence, or learning of a trauma that happened to a close relative or friend). The provider then asked questions such as “Which of these events currently gets in the way of your life the most?”, “Which one of these events do you find yourself having the most upsetting and unwanted thoughts about lately?”, and “What is the event that makes you most distressed or upset when it comes into your head in thoughts or flashbacks, or when you are reminded of it?” to help the participant establishing which experiences they personally found most, second, and third most distressing at the time of treatment.

To determine if any patterns existed among the reported top three traumatic experiences in this sample, the analysis examined rates of various trauma types endorsed by participants in the IOP-PE arm. Two behavioral health care providers experienced in treatment of military personnel with PTSD independently reviewed each reported trauma and classified it using the categorization schemes described below. Determinations were made based on clinical expertise and evaluation of descriptions of each traumatic experience available via retrospective review of clinical notes collected by study providers prior to treatment. These raters discussed any discrepant classifications until coming to consensus. If consensus could not be reached, discrepant responses were reviewed for a final determination by a third rater who served as a treatment provider for the clinical trial and had extensive, first-hand knowledge of the participant’s cases.

Five trauma classification schemes were utilized, including one categorical and four dichotomous rating systems. Events were categorically rated using the scheme for classifying traumatic military events developed by Litz and colleagues (referred to herein as the “Litz classification”; Stein et al., 2012). This system assigns traumatic experiences to one of six categories: life threat to self, life threat to others, aftermath of violence, traumatic loss, moral injury by self, and moral injury by others. The four dichotomous classifications were (1) sexual trauma vs. non-sexual trauma, (2) military-related vs. non-military related trauma, (3) combat-related vs. non-combat related trauma, and (4) trainability vs. futility traumatic experiences (De Rond & Lok, 2016; Macia et al., 2020; Martin et al., 2000). Trainability events were defined as ones for which service members likely had previous military training specifically addressing steps to take when faced with these events and therefore they could potentially assist with resolving or mitigating negative outcomes. These events are characterized by service members reasonably perceiving that they had the skills and ability to help in whatever way the situation required, regardless of the actual outcome. This could include a medic losing a patient to wounds they felt they should have been able to treat based on their training and experience level. Futility events were ones that could be reasonably perceived as outside of service members’ control. This could include events such as witnessing the instantaneous death of a fellow service member in an improvised explosive device (IED) blast. These events were characterized by a reasonable perception that, regardless of training, there was not anything more that could have been done in the moment to improve outcomes (Hale et al., 2021). Appendix A, Table A1 includes definitions for each of these constructs.

### Data Analytic Strategy

Prevalence rates were calculated for each trauma type designated by the participant as (1) the most distressing (primary/index event), (2) the second most distressing, (3) the third or least distressing, and (4) the overall sum of all reported events. Different (vs. similar) rates of trauma types across the three identified events were explored to determine if certain trauma categories were reported at notably different rates for most, second most, and third most distressing events. Additionally, the relationship between demographic and military characteristics and trauma type were examined using chi-square tests for nominal variables and general linear models for continuous variables. Select variables were collapsed where conceptually appropriate to maximize adequate cell sizes and equal distribution across characteristic groups. Effect sizes (Standardized adjusted residuals; Cramer’s *V*) were also calculated to indicate the magnitude of significant effects. A standardized adjusted residual (*z*) greater than ±1.96 was interpreted as an observed frequency that significantly differed from what was expected by chance. Effect sizes of *V* = 0.07, 0.21, and 0.35 were interpreted as small, medium, and large, respectively (Sun et al., 2010).

## 3. Results

The sample of U.S. military service members and veterans (*N* = 110) was diverse, with 38% (42/110) of participants identifying as Non-Hispanic White, 28% (31/110) identifying as African American, 26% (29/110) identifying as Hispanic, and 4% (4/110) identifying as “other race”. Participants were primarily men (80%; 88/110) and married (72%; 79/110), with an average age of 39 years old. Most of the sample was on active duty (68%; 75/110), currently served or had served in the Army (80%; 88/110), and reported an average of 15 years of service (see Table 1).

Of the 110 participants, 80% (88/110) identified and ranked their three most traumatic experiences, 8% (9/110) reported and ranked only two traumatic experiences, and 12% (13/110) reported only a single traumatic experience. Traumatic experiences that could not be classified due to lack of available information and items that did not fit into the Litz classification system (*n* = 3) were excluded from analyses.

### 3.1. Prevalence of Trauma Types

#### 3.1.1. All Traumatic Experiences

**Figure 1:**
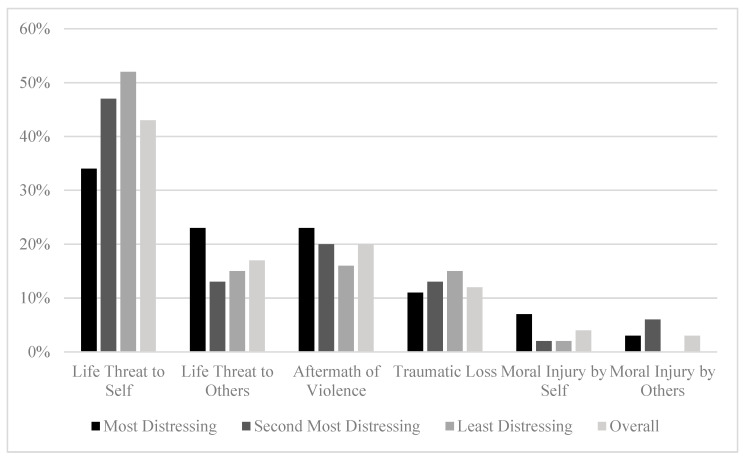
Litz Trauma Categorization Distribution Percentages Across Top Three.

**Figure 2:**
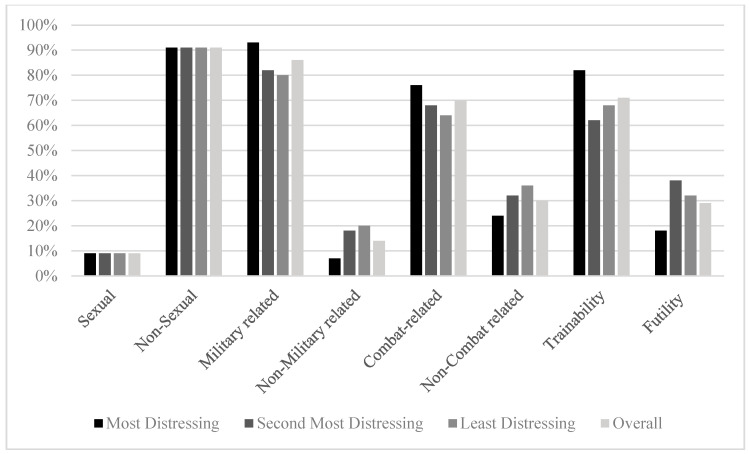
Dichotomous Trauma Categorization Distribution Percentages Across Top Three Traumatic Experiences.

When examining all traumatic experiences (most distressing, second most distressing, third most distressing) according to the six Litz categories, Life Threat to Self was reported most often (43%; 119/274; see Figure 1). Most traumatic experiences were dichotomously classified as non-sexual (91%; 268/295), military-related (86%; 248/290), and combat-related (70%; 202/290; see Figure 1). Additionally, 71% (210/295) of reported traumatic experiences were classified as trainability, rather than futility traumatic experiences. For a complete account of trauma categorizations across the top three traumatic experiences, see Figure 1 and Figure 2.

Traumatic Experiences

#### 3.1.2. Most Distressing Trauma

Of the most distressing traumatic experiences, 34% (35/104) were classified as Life Threat to Self (see Figure 1). Similar patterns as the overall classifications were seen among the dichotomous primary traumatic experiences (see Figure 2).

#### 3.1.3. Second Most Distressing Trauma

Percentage of items classified as Life Threat to Self was higher among the second most distressful events compared with most distressing events (34% [35/104] compared to 47% [41/88]; see Figure 1). Rates of non-sexual versus sexual traumatic experiences were consistent (see Figure 2). Military-related, combat-related, and trainability events were lower compared to primary traumatic experiences, with the largest percentage difference seen in trainability traumatic experiences, accounting for 62% (60/97) of all secondary traumatic experiences, compared to 82% (90/110) of primary traumatic experiences.

#### 3.1.4. Third Most Distressing Trauma

Finally, Life Threat to Self accounted for over half of all third most traumatic experiences (52%; 43/82). Non-sexual traumatic experiences again accounted for most of the reported traumatic experiences (91%; 80/88). Military- and combat-related traumatic experiences were lower compared to the second most distressing traumatic experiences, accounting for 80% (68/85) and 64% (54/85) of the third most traumatic experiences, respectively. Lastly, trainability traumatic experiences accounted for 68% (60/88) of third most traumatic experiences.

### 3.2. Demographic and Military Characteristics and Trauma Types

Relationships among all demographic and military characteristics and trauma categorizations are presented in Appendix B, Table A2, Table A3, Table A4 and Table A5. Notably, gender was significantly related to all categorizations when looking at all traumatic experiences combined, and most categorizations when broken down by top three traumatic experiences. Women were highly over-represented among sexual traumatic experiences across their most (*z* = 5.0, *V* = 0.474), second most (*z* = 4.1, *V* = 0.413), and third most (*z* = 5.3, *V* = 0.568) distressing experiences compared to men, as well as when examining all traumatic experiences combined (*z* = 8.3, *V* = 0.482). Men were more likely than women to report combat-related and military-related traumatic experiences for their most (*z* = 4.9, *V* = 0.469; *z* = 4.2, *V* = 0.398, respectively) and second most distressing events (*z* = 2.6, *V* = 0.263; *z* = 2.1, *V* = 0.214, respectively), as well as for all traumatic experiences combined (*z* = 5.3, *V* = 0.312). Finally, gender was also associated with trainability/futility categorizations for the most distressing trauma and all traumatic experiences combined, with female service members more likely to report futility traumatic experiences (*z* = 4.9, *V* = 0.471; *z* = 4.7, *V* = 0.272).

After gender, military occupational specialty category (e.g., type of combat work) and number of deployments were significantly associated with the most categories across the top three most traumatic experiences and all experiences combined. Time in military, age, marital status, branch, and active duty status (vs. veteran status) also demonstrated relationships with various traumatic experience categorizations. No significant relationships were found between traumatic experience categorizations and rank, race, or education. Significant findings across all military and demographic characteristics are presented in bold text in Appendix B, Table A2, Table A3, Table A4 and Table A5.

## 4. Discussion

This study evaluated and categorized the top three traumatic experiences identified by U.S. active duty service members and veterans seeking treatment for PTSD according to five trauma classification schemes and then calculated the percentages of different trauma types. For the categorical Litz classification, Life Threat to Self was consistently the most prevalent trauma type across all top three traumatic experiences, accounting for approximately one third of the most distressing traumatic experiences and half of the second and third most distressing traumatic experiences. Notably, Life Threat to Self was over-represented among female service members and underrepresented among combat arms personnel. It may be that combat arms personnel had been trained for and were well prepared for the potential threats to their lives during a combat deployment and were thereby less distressed when they occurred. Conversely, most female military personnel serve as noncombatants and may have less training and preparation for life threats that can occur during a deployment. Moral Injury by Self and Moral Injury by Others were consistently the least represented categories, accounting for less than 10% of the most, second most, and third most distressing traumatic experiences. The relative infrequency of these moral injury-related experiences reported in this sample is not necessarily indicative of lower PTSD symptom severity associated with these experiences. It is more likely that events involving perceived or actual life threat simply occur at a higher frequency in a deployed setting than morally injurious events. PTSD severity as it relates to each traumatic experience is outside the scope of these analyses, and has been described elsewhere (Guina et al., 2018; Jakob et al., 2017).

Combat trauma categorizations made up 64–76% of all traumatic experiences and were associated with various demographic and military characteristics, including gender, active duty status, branch, occupational specialty, and number of deployments. Combat trauma was overrepresented in this sample among male service members, participants who had deployed three times, Army members, veterans, and combat arms personnel. Similarly, military-related traumatic experiences accounted for a large majority of experiences in this sample (80–93%), with overrepresentation among male service members and combat arms.

Trainability traumatic experiences made up 62–82% of all traumatic experiences in this sample. Trainability traumatic experiences were overrepresented among married service members, male service members, combat arms, and people who had deployed three times. It was also associated with more years in the military compared to futility traumatic experiences. The high rate of participants seeking treatment for PTSD related to events for which service members are trained could indicate areas of interest for improved or additional predeployment training. Alternatively, trainability traumatic experiences may be overrepresented in this sample due to a potential relationship between feelings of trauma-related guilt over the service member not being able to prevent an event for which they have been trained (Norman et al., 2018).

Gender played an important role across all top three trauma categories, with women highly overrepresented among sexual traumatic experiences and underrepresented among military- and combat-related traumatic experiences. Though sexual trauma made up only 9% (27/295) of all experiences in this sample, it made up 38% (21/55) of the traumatic experiences reported by female service members. Traumatic experiences reported by women were also more likely to be categorized as Life Threat to Self. These findings are consistent with previous research showing that while female service members are exposed to lower rates of combat trauma, they are at substantially higher risk of military sexual trauma than their male counterparts (Chaumba & Bride, 2010). Although sexual trauma made up a small portion of the total study sample, the large gender differences seen here, coupled with prior research demonstrating more severe PTSD symptom severity related to sexual trauma versus other types of traumatic experiences, highlight the disproportionate impact sexual trauma has on the mental health of female service members (Jakob et al., 2017).

### Limitations

For the purpose of this analysis, only a single Litz category was selected for each traumatic experience. However, the six categories defined by this classification system are not mutually exclusive, and therefore assigning a traumatic experience to one category may not fully characterize the event. Furthermore, the Litz categorizations are often identified by patients, rather than by providers, and patient/provider agreement may be poor. Given the retrospective nature of this secondary data analysis, the researchers were unable to obtain Litz categorizations directly from participants, and descriptions of the traumatic experiences were, in some cases, vague. However, efforts were made to accurately classify each traumatic experience through independent ratings by multiple behavioral health professionals, some of whom were involved in patient care during data collection of the parent study, as described above. Finally, inclusion in this study required participants to report at least one combat-related Criterion A event related to their PTSD symptoms. This requirement could have impacted the variability of classification rates presented herein by excluding service members and veterans who attribute their PTSD symptoms exclusively to non-combat related events. However, this limitation was also potentially partially mitigated by the opportunity to discuss top three traumatic events, rather than a single event.

## 5. Conclusions

This study aimed to categorize and evaluate patterns of top three most traumatic experiences reported by U.S. service members with PTSD. The results of this study highlight that, although direct combat-related experiences involving Life Threat to Self were the most commonly reported type of traumatic experience in this sample, there was noteworthy variability in types of traumatic experience across top three traumas, especially when evaluating certain subpopulations, such a female service members. This is important because traditional trauma-focused treatments for PTSD solely address the single most traumatic experience reported by service members—which, as seen in this sample is often a combat-related life threat event. This approach may fail to address other critical Criterion A events, such as events related sexual traumas, which are reported less frequently overall but disproportionately impact female service members and have been shown to result in worse PTSD symptom severity than other types of traumatic experience (Jakob et al., 2017). Furthermore, some types of traumatic experiences may go underreported in traditional trauma-focused treatments. When asked to report their most distressing of multiple traumatic experience, service members may be compelled to disclose combat-related life threat in lieu of instances of moral injury due to potential guilt and self-condemnation or instances of sexual trauma due to distrust in the military institution and fear of the repercussions of disclosure (Dardis et al., 2018; Griffin et al., 2019; Vermetten & Jetly, 2018). Addressing the top three traumatic experiences during PTSD treatment may provide service members with more opportunities to acclimate to treatment and develop trust with their therapist before examining the details of their most distressing traumatic events. Additionally, it may provide patients with the opportunity to address diverse experiences (e.g., a combat-related experience and a sexual trauma), which may in turn better address different PTSD symptom clusters associated with certain categories of traumatic experiences (Guina et al., 2018). Addressing top three most traumatic experiences might not be beneficial to all U.S. service members with PTSD, however, providers could consider offering patients the choice between the top three versus single most traumatic experience. This may lead to better treatment outcomes as it would allow the patient to decide which events they think are relevant to their symptoms and which they feel comfortable addressing. Research evaluating the relationship between patient choice and PTSD treatment outcomes has demonstrated that patients see greater improvements when they get a say in their treatment plan (Le et al., 2018).

Many trauma victims in the U.S. general population experience multiple traumas of various types in their lifetime (Kilpatrick et al., 2013). This is especially true of the U.S. military population, as supported herein, with a majority of participants identifying multiple traumatic experiences and wide diversity in traumatic experience classification when taking demographic and military characteristics into account (Dedert et al., 2009). Future research and clinical practice should account for the unique experiences of U.S. service members and exposure to multiple diverse, potentially traumatic experiences and consider adapting treatments to address a patient’s individual trauma symptomatology. These findings also highlight the ongoing prevalence of sexual trauma among female service members. Continued efforts must be made to prevent sexual assault and harassment within the military, with an emphasis on protecting and empowering female service members.

## Figures and Tables

**Table 1:** Demographic and Military Characteristics.

Characteristic	Participant (*N* = 110), No. (%)
Age, mean (*SD*), y	39.40 (7.22)
Gender	
Women	22 (20)
Men	88 (80)
Married	79 (72)
Race and ethnicity	
African American	31 (28)
Hispanic	29 (26)
Non-Hispanic White	42 (38)
Other	4 (4)
Education	
≤Some college	48 (44)
≥Associate degree	62 (56)
Active duty	75 (68)
Army	88 (80)
Time in military, mean (*SD*), y	15.22 (6.51)
Occupational Specialty	
Combat arms	44 (40)
Combat support	31 (28)
Combat service support	34 (31)
Deployments, No.	
1	27 (25)
2	33 (30)
3	23 (21)
≥4	26 (24)

## Data Availability

The data presented in this study are maintained at The University of Texas Health Science Center at San Antonio in the STRONG STAR Repository. Requests for access to the data as well as for materials and the analysis code also can be emailed to repository@strongstar.org.

## Abbreviations

The following abbreviations are used in this manuscript:

CAPS-5	Clinician-Administered PTSD Scale for DSM-5
DSM-5	Diagnostic and Statistical Manual for Mental Disorders
IED	Improvised Explosive Device
IOP	Intensive Outpatient Program
PE	Prolonged Exposure
PTSD	Posttraumatic Stress Disorder
SD	Standard Deviation

## Appendix A

**Table A1:** Trauma Category Classification Definitions.

Category	Operational Definition
**Litz classification** **(Stein et al., 2012)**	
Life Threat to Self	Personal exposure to the threat of death or actual or threatened serious injury
Life Threat to Others	Personal exposure to the actual or threatened death of others
Aftermath of Violence	Personal exposure to grotesque or haunting images, sounds, or smells of dead or severely injured humans or animals
Traumatic Loss	Witnessed or learned about the death of a family member, friend, or unit member
Moral Injury by Self	Committing an act that is perceived to be a gross violation of moral or ethical standards (e.g., killing or injuring others, rape, atrocities). A service member who nearly committed these acts could also experience moral injury.
Moral Injury by Others	Witnessing or being victim of an act that is perceived to be a gross violation of moral or ethical standards (e.g., killing or injuring civilians, rape, atrocities, betrayal). Events can also be indirectly experienced (i.e., learned about) if they are directly relevant to the individual.
Not within Litz classification	Experiences that do not fit within the definitions of the six categories defined above.
**Sexual trauma classification**	
Sexual trauma	Traumatic experiences related to sexual trauma/assault in a deployed or non-deployed setting. Can include childhood sexual trauma, military sexual trauma, etc.
Non-sexual trauma	Factors not related to sexual trauma/assault.
**Military trauma classification**	
Military-related	Factors related to military service, whether combat related or not. Examples include traumatic experiences that occur during training, military sexual trauma, combat related trauma, negative interactions with civilians in a deployed setting, etc.
Non-military-related	Factors not related to military service, combat, military training, etc. This includes events that occur in a civilian setting, such as childhood sexual assault, motor vehicle accidents, non-military related death of a loved one, etc.
**Combat trauma classification**	
Combat-related	Factors directly (confronting enemy combatants) or indirectly (providing care to a service member injured in combat, mortuary services, etc.) related to combat
Noncombat-related	Factors not related to combat, such as military sexual assault, motor vehicle accidents in a civilian/non-deployed setting, etc.
**Trainability classification** **(Hale et al., 2021)**	
Trainability	Factors that could be anticipated and prepared for via training, that the service member reasonably feels they could have mitigated. This can include medically treating fellow service members for common wounds.
Futility	Events that are reasonably perceived as outside of the service member’s control. This can include events such as trying to medically treat a service member who was too badly injured to be stabilized, events that the service member heard about but was not physically present for (e.g., death of a loved one), etc.

## Appendix B

**Table A2:** Relationships Between Trauma Categorizations and Demographic/Military Characteristic Across All Reported Traumatic Experiences (*N* = 295).

	Life Treat to Self (*n* = 119)	Life Threat to Others (*n* = 47)	Aftermath of Violence (*n* =55)	Traumatic Loss (*n* = 34)	Moral Injury by Self (*n* = 11)	Moral Injury by Others (*n* = 8)	Sexual Trauma (*n* = 27)	Military-Related (*n* = 248)	Combat-Related (*n* = 202)	Trainability (*n* = 210)
	% (*n*)									
**Age (*F*)**	0.96						**4.71 ***	0.07	0.87	2.1
**Gender (χ^2^)**	**22.9 *****						**68.52 *****	**7.46 ****	**28.24 *****	**21.82 *****
Women	**31.9 (38) ^a^**	17 (8)	**5.5 (3) ^b^**	**5.9 (2) ^b^**	18.2 (2)	12.5 (1)	**77.8 (21) ^a^**	**15.7 (39) ^b^**	**10.9 (22) ^b^**	**11.9 (25) ^b^**
Men	**68.1 (81) ^b^**	83 (39)	**94.5 (52) ^a^**	**94.1 (32) ^a^**	81.8 (9)	87.5 (7)	**22.2 (6) ^b^**	**84.3 (209) ^a^**	**89.1 (180) ^a^**	**88.1 (185) ^a^**
**Marital Status (χ^2^)**	6.66						**4.95 ***	1.89	0.73	1.25
Married	73.1 (87)	85.1 (40)	76.4 (42)	67.6 (23)	54.5 (6)	62.5 (5)	**55.6 (15) ^b^**	73.4 (182)	75.2 (152)	75.7 (159)
Not Married	26.9 (32)	14.9 (7)	23.6 (13)	32.4 (11)	45.5 (5)	37.5 (3)	**44.4 (12) ^a^**	26.6 (66)	24.8 (50)	24.3 (51)
**Race and ethnicity (χ^2^)**	13.38						4.49	0.39	1.56	3.94
African American	32.2 (37)	37.8 (17)	28.3 (15)	18.8 (6)	9.1 (1)	37.5 (3)	44.4 (12)	29.5 (71)	27.8 (55)	26.8 (55)
Hispanic	37.4 (43)	42.2 (19)	41.5 (22)	43.8 (14)	63.6 (7)	50 (4)	25.9 (7)	40.7 (98)	41.9 (83)	43.4 (89)
Non-Hispanic White	26.1 (30)	17.8 (8)	30.2 (16)	34.4 (11)	18.2 (2)	12.5 (1)	29.6 (8)	27 (65)	27.8 (55)	26.8 (55)
Other	4.3 (5)	2.2(1)	0 (0)	3.1 (1)	9.1 (1)	0 (0)	0 (0)	2.9 (7)	2.5 (5)	2.9 (6)
**Education (χ^2^)**	3.41						0	0.73	3.77	2
≤Some college	39.5 (47)	44.7 (21)	52.7 (29)	41.2 (14)	54.5 (6)	50 (4)	44.4 (12)	45.2 (112)	47.5 (96)	46.7 (98)
≥Associate degree	60.5 (72)	55.3 (26)	47.3 (26)	58.8 (20)	45.5 (5)	50 (4)	55.6 (15)	54.8 (136)	52.5 (106)	53.3 (11)
**Status (χ^2^)**	3.7						2.12	3.3	2.77	0.38
Active duty	70.6 (84)	63.8 (30)	76.4 (42)	73.5 (25)	72.7 (8)	50 (4)	81.5 (22)	66.9 (166)	66.3 (134)	68.1 (143)
Veteran	29.4 (35)	36.2 (17)	23.6 (13)	26.5 (9)	27.3 (3)	50 (4)	18.5 (5)	33.1 (82)	33.7 (67)	31.9 (67)
**Branch (χ^2^)**	7.56						0.16	0.28	3.36	0.04
Army	75.6 (90)	78.7 (37)	87.3 (48)	91.2 (31)	81.8 (9)	62.5 (5)	77.8 (21)	79.8 (198)	83.2 (168)	81 (170)
Other	24.4 (29)	21.3 (10)	12.7 (7)	8.8 (3)	18.2 (2)	37.5 (3)	22.2 (6)	20.2 (50)	16.8 (34)	19 (40)
**Time in Military (*F*)**	1.39						2.43	0.17	0.62	**4.73 ***
**Occupational Specialty (χ^2^)**	6.7						**20.48 *****	**8.68 ***	**10.15 ****	**9.22 ****
Combat arms	36.2 (42)	36.2 (17)	41.8 (23)	55.9 (19) ^a^	36.4 (4)	50 (4)	**4 (1) ^b^**	**44.9 (110) ^a^**	**47.3 (95) ^a^**	**46.9 (98) ^a^**
Combat support	26.7 (31)	34 (16)	25.5 (14)	20.6 (7)	36.4 (4)	25 (2)	28 (7)	**24.9 (61) ^b^**	26.4 (53)	24.9 (52)
Combat service support	37.1 (43)	29.8 (14)	32.7 (18)	23.5 (8)	27.3 (3)	25 (2)	**68 (17) ^a^**	30.2 (74)	**26.4 (53) ^b^**	**28.2 (59) ^b^**
**Rank (χ^2^)**	3.3						0.06	0.38	0.22	0.67
Enlisted	86.6 (103)	89.4 (42)	87.3 (48)	79.4 (27)	90.9 (10)	100 (8)	88.9 (24)	87.1 (216)	86.6 (175)	87.1 (183)
Officer	13.4 (16)	10.6 (5)	12.7 (7)	20.6 (7)	9.1 (1)	0 (0)	11.1 (3)	12.9 (32)	13.4 (27)	12.9 (27)
**Deployments, No. (χ^2^)**	23.2						**9.73 ***	6.39	**8.24 ***	**13.13 ****
1	22.4 (26)	27.7 (13)	16.4 (9)	26.5 (9)	9.1 (1)	0 (0)	24 (6)	20.4 (50) ^b^	**19.4 (39) ^b^**	**18.2 (38) ^b^**
2	35.3 (41)	31.9 (15)	27.3 (15)	20.6 (7)	45.5 (5)	50 (4)	**56 (14) ^a^**	32.2 (79)	29.4 (59)	31.1 (65)
3	23.3 (27)	19.1 (9)	27.3 (15)	11.8 (4)	45.5 (5)	37.5 (3)	12 (3)	23.3 (57)	**25.9 (52) ^a^**	**26.3 (55) ^a^**
≥4	19 (22)	21.3 (10)	29.1 (16)	41.2 (14) ^a^	0 (0)	12.5 (1)	8 (2)	24.1 (59)	25.4 (51)	24.4 (51)

Due to missing data, some categories may not represent 100% of the total sample of reported traumatic experiences. Significant findings presented in bold. * < 0.05, ** *p* < 0.01, *** *p* < 0.001. ^a^ indicates an adjusted residual (*z*) greater than 1.96. ^b^ indicates an adjusted residual (*z*) less than −1.96.

**Table A3:** Relationships Between Trauma Categorizations and Demographic/Military Characteristic Across Most Distressing Traumatic Experiences (*N* = 110).

	Life Treat to Self (*n* = 35)	Life Threat to Others (*n* = 24)	Aftermath of Violence (*n* =24)	Traumatic Loss (*n* = 11)	Moral Injury by Self (*n* = 7)	Moral Injury by Others (*n* = 3)	Sexual Trauma (*n* = 10)	Military-Related (*n* = 101)	Combat-Related (*n* = 83)	Trainability (*n* = 90)
	% (*n*)									
**Age (*F*)**	0.74						**4.62 ***	0.01	2.68	**4.36 ***
**Gender (χ^2^)**	5.87						**24.75 *****	**17.24 *****	**24.02 *****	**24.44 *****
Women	28.6 (10)	29.2 (7)	8.3 (2)	9.1 (1)	14.3 (1)	33.3 (1)	**80 (8) ^a^**	**14.9 (15) ^b^**	**9.6 (8) ^b^**	**11.1 (10) ^b^**
Men	71.4 (25)	70.8 (17)	91.7 (22)	90.9 (10)	85.7 (6)	66.7 (2)	**20 (2) ^b^**	**85.1 (86) ^a^**	**90.4 (75) ^a^**	**88.9 (80) ^a^**
**Marital Status (χ^2^)**	3.16						2.59	0.03	0.64	0.56
Married	74.3 (26)	83.3 (20)	66.7 (16)	63.6 (7)	57.1 (4)	66.7 (2)	50 (5)	72.3 (73)	73.5 (61)	73.3 (66)
Not Married	25.7 (9)	16.7 (4)	33.3 (8)	36.4 (4)	42.9 (3)	33.3 (1)	50 (5)	27.7 (28)	26.5 (22)	26.7 (24)
**Race and ethnicity (χ^2^)**	12.97						3.26	0.32	0.9	2.54
African American	32.4 (11)	39.1 (9)	30.4 (7)	0 (0)b	14.3 (1)	33.3 (1)	50 (5)	29.6 (29)	29.6 (24)	27.3 (24)
Hispanic	29.4 (10)	21.7 (5)	30.4 (7)	40 (4)	14.3 (1)	0 (0)	10 (1)	27.6 (27)	29.6 (24)	29.5 (26)
Non-Hispanic White	32.4 (11)	34.8 (8)	39.1 (9)	60 (6)	57.1 (4)	66.7 (2)	40 (4)	38.8 (38)	37 (30)	38.6 (34)
Other	5.9 (2)	4.3 (1)	0 (0)	0 (0)	14.3 (1)	0 (0)	0 (0)	4.1 (4)	3.7 (3)	4.5 (4)
**Education (χ^2^)**	2.94						0.06	0.11	3.65	1.85
≤Some college	42.9 (15)	50 (12)	45.8 (11)	27.3 (3)	28.6 (2)	66.7 (2)	40 (4)	43.6 (44)	48.2 (40)	46.7 (42)
≥Associates degree	57.1 (20)	50 (12)	54.2 (13)	72.7 (8)	71.4 (5)	33.3 (1)	60 (6)	56.4 (57)	51.8 (43)	53.3 (48)
**Status (χ^2^)**	1.76						0.71	0.2	**3.98 ***	0.52
Active duty	71.4 (23)	58.3 (14)	70.8 (17)	72.7 (8)	57.1 (4)	66.7 (2)	80 (8)	67.3 (68)	**63.9 (53) ^b^**	66.7 (60)
Veteran	28.6 (10)	41.7 (10)	29.2 (7)	27.3 (3)	42.9 (3)	33.3 (1)	20 (2)	32.7 (33)	**36.1 (30) ^a^**	33.3 (30)
**Branch (χ^2^)**	2.7						0	0.12	**4.41 ***	1.53
Army	77.1 (27)	70.8 (17)	83.3 (20)	90.9 (10)	85.7 (6)	66.7 (2)	80 (8)	80.2 (81)	**84.3 (70) ^a^**	82.2 (74)
Other	22.9 (8)	29.2 (7)	16.7 (4)	9.1 (1)	14.3 (1)	33.3 (1)	20 (2)	19.8 (20)	**15.7 (13) ^b^**	17.8 (16)
**Time in Military (*F*)**	0.42						2.12	0.07	1.44	**3.99 ***
**Occupational Specialty (χ^2^)**	4.95						**6.71 ***	**10.06 ****	5.83	**8.85 ***
Combat arms	35.3 (12)	33.3 (8)	37.5 (9)	54.5 (6)	42.9 (3)	66.7 (2)	**0 (0) ^b^**	**44 (44) ^a^**	47 (39) ^a^	**46.7 (42) ^a^**
Combat support	26.5 (9)	33.3 (8)	29.2 (7)	18.2 (2)	42.9 (3)	33.3 (1)	44.4 (4)	**25 (25) ^b^**	25.3 (21)	**24.4 (22) ^b^**
Combat service support	38.2 (13)	33.3 (8)	33.3 (8)	27.3 (3)	14.3 (1)	0 (0)	55.6 (5)	31 (31)	27.7 (23)	28.9 (26)
**Rank (χ^2^)**	4.65						0.12	0.01	0.08	0.27
Enlisted	91.4 (32)	91.7 (22)	79.2 (19)	72.7 (8)	85.7 (6)	100 (3)	90 (9)	86.1 (87)	86.7 (72)	85.6 (77)
Officer	8.6 (3)	8.3 (2)	20.8 (5)	27.3 (3)	14.3 (1)	0 (0)	10 (1)	13.9 (14)	13.3 (11)	14.4 (13)
**Deployments, No. (χ^2^)**	14.62						3.29	1.42	6.69	5.35
1	23.5 (8)	29.2 (7)	29.2 (7)	18.2 (2)	14.3 (1)	0 (0)	33.3 (3)	24 (24)	21.7 (18)	22.2 (20)
2	29.4 (10)	29.2 (7)	29.2 (7)	36.4 (4)	57.1 (4)	33.3 (1)	44.4 (4)	30 (30)	26.5 (22)	27.8 (25)
3	20.6 (7)	25 (6)	16.7 (4)	0 (0)	28.6 (2)	66.7 (2) ^a^	22.2 (2)	21 (21)	22.9 (19)	23.3 (21)
≥4	26.5 (9)	16.7 (4)	25 (6)	45.5 (5)	0 (0)	0 (0)	0 (0)	25 (25)	28.9 (24) ^a^	26.7 (24)

Due to missing data, some categories may not represent 100% of the total sample of reported traumatic experiences. Significant findings presented in bold. * < 0.05, ** *p* < 0.01, *** *p* < 0.001. ^a^ indicates an adjusted residual (*z*) greater than 1.96. ^b^ indicates an adjusted residual (*z*) less than −1.96.

**Table A4:** Relationships Between Trauma Categorizations and Demographic/Military Characteristic Across Second Most Distressing Traumatic Experiences (*N* = 97).

	Life Treat to Self (*n* = 41)	Life Threat to Others (*n* = 11)	Aftermath of Violence (*n* = 18)	Traumatic Loss (*n* = 11)	Moral Injury by Self (*n* = 2)	Moral Injury by Others (*n* = 5)	Sexual Trauma (*n* = 9)	Military-Related (*n* = 79)	Combat-Related (*n* = 65)	Trainability (*n* = 60)
	% (*n*)									
**Age (*F*)**	0.67						1.5	1.62	0	0.01
**Gender (χ^2^)**	**15.31 ****						**16.57 *****	**4.38 ***	**6.65 ***	3.74
Women	**36.6 (15) ^a^**	9.1 (1)	0 (0) ^b^	9.1 (1)	0 (0)	0 (0)	**66.7 (6) ^a^**	**13.9 (11) ^b^**	**89.2 (58) ^a^**	11.7 (7)
Men	**63.4 (26) ^b^**	90.9 (10)	100 (18) ^a^	90.9 (10)	100 (2)	100 (5)	**33.3 (3) ^b^**	**86.1 (68) ^a^**	**10.8 (7) ^b^**	88.3 (53)
**Marital Status (χ^2^)**	6.03						1.81	0.21	3.82	**4.55 ***
Married	70.7 (29)	90.9 (10)	83.3 (15)	54.5 (6)	100 (2)	60 (3)	55.6 (5)	75.9 (60)	80 (52) ^a^	**81.7 (49) ^a^**
Not Married	29.3 (12)	9.1 (1)	16.7 (3)	45.5 (5)	0 (0)	40 (2)	44.4 (4)	24.1 (19)	20 (13) ^b^	**18.3 (11) ^b^**
**Race and ethnicity (χ^2^)**	8.69						0.44	2.28	1.7	2.79
African American	35.9 (14)	40 (4)	16.7 (3)	36.4 (4)	0 (0)	40 (2)	33.3 (3)	28.6 (22)	26.6 (17)	27.1 (16)
Hispanic	23.1 (9)	10 (1)	33.3 (6)	27.3 (3)	50 (1)	20 (1)	33.3 (3)	28.6 (22)	29.7 (19)	25.4 (15)
Non-Hispanic White	38.5 (15)	50 (5)	50 (9)	27.3 (3)	50 (1)	40 (2)	33.3 (3)	41.6 (32)	42.2 (27)	45.8 (27)
Other	2.6 (1)	0 (0)	0 (0)	9.1 (1)	0 (0)	0 (0)	0 (0)	1.3 (1)	1.6 (1)	1.7 (1)
**Education (χ^2^)**	5.5						0.51	0.75	0.47	1.02
≤ Some college	36.6 (15)	45.5 (5)	61.1 (11)	45.5 (5)	100 (2)	40 (2)	55.6 (5)	46.8 (37)	46.2 (30)	48.3 (29)
≥ Associates degree	63.4 (26)	54.5 (6)	38.9 (7)	54.5 (6)	0 (0)	60 (3)	44.4 (4)	53.2 (42)	53.8 (35)	51.7 (31)
**Status (χ^2^)**	5.55						0.43	2.03	0.88	0.01
Active duty	70.7 (29)	81.8 (9)	77.8 (14)	54.5 (6)	100 (2)	40 (2)	77.8 (7)	64.6 (51)	64.6 (42)	68.3 (41)
Veteran	29.3 (12)	18.2 (2)	22.2 (4)	45.5 (5)	0 (0)	60 (3)	22.2 (2)	35.4 (28)	35.4 (23)	31.7 (19)
**Branch (χ^2^)**	8.4						0.09	2.25	0.1	0.22
Army	75.6 (31)	81.8 (9)	94.4 (17)	100 (11)	50 (1)	60 (3)	77.8 (7)	78.5 (62)	81.5 (53)	80 (48)
Other	24.4 (10)	18.2 (2)	5.6 (1)	0 (0)	50 (1)	40 (2)	22.2 (2)	21.5 (17)	18.5 (12)	20 (12)
**Time in Military (*F*)**	0.41						1.6	0.93	0.01	0.26
**Occupational Specialty (χ^2^)**	12.08						**8.1 ***	2.95	4.1	1.35
Combat arms	25 (10) ^b^	45.5 (5)	55.6 (10)	63.6 (7)	50 (1)	40 (2)	**0 (0) ^b^**	44.9 (35)	46.2 (30)	45 (27)
Combat support	30 (12)	36.4 (4)	27.8 (5)	9.1 (1)	50 (1)	20 (1)	25 (2)	25.6 (20)	27.7 (18)	25 (15)
Combat service support	45 (18) ^a^	18.2 (2)	16.7 (3)	27.3 (3)	0 (0)	40 (2)	**75 (6) ^a^**	29.5 (23)	26.2 (17) ^b^	30 (18)
**Rank (χ^2^)**	3.17						1.4	0.83	3.6	1.0
Enlisted	85.4 (35)	90.9 (10)	88.9 (16)	72.7 (8)	100 (2)	100 (5)	100 (9)	86.1 (68)	83.1 (54)	85 (51)
Officer	14.6 (6)	9.1 (1)	11.1 (2)	27.3 (3)	0 (0)	0 (0)	0 (0)	13.9 (11)	16.9 (11)	15 (9)
**Deployments, No. (χ^2^)**	15.87						5.43	0.53	1.14	1.47
1	25 (10)	18.2 (2)	11.1 (2)	36.4 (4)	0 (0)	0 (0)	25 (2)	23.1 (18)	24.6 (16)	20 (12)
2	35 (14)	36.4 (4)	27.8 (5)	9.1 (1)	0 (0)	60 (3)	62.5 (5) ^a^	30.8 (24)	27.7 (18)	31.7 (19)
3	22.5 (9)	27.3 (3)	22.2 (4)	27.3 (3)	100 (2) ^a^	20 (1)	12.5 (1)	21.8 (17)	23.1 (15)	25 (15)
≥4	17.5 (7)	18.2 (2)	38.9 (7)	27.3 (3)	0 (0)	20 (1)	0 (0) ^b^	24.4 (19)	24.6 (16)	23.3 (14)

Due to missing data, some categories may not represent 100% of the total sample of reported traumatic experiences. Significant findings presented in bold. * < 0.05, ** *p* < 0.01, *** *p* < 0.001. ^a^ indicates an adjusted residual (*z*) greater than 1.96. ^b^ indicates an adjusted residual (*z*) less than −1.96.

**Table A5:** Relationships Between Trauma Categorizations and Demographic/Military Characteristic Across Third Most Distressing Traumatic Experiences (*N* = 88).

	Life Treat to Self (*n* = 43)	Life Threat to Others (*n* = 12)	Aftermath of Violence (*n* =13)	Traumatic Loss (*n* = 12)	Moral Injury by Self (*n* = 2)	Moral Injury by Others (*n* = 0)	Sexual Trauma (*n* = 8)	Military-Related (*n* = 68)	Combat-Related (*n* = 54)	Trainability (*n* = 60)
	% (*n*)									
**Age (*F*)**	0.11						0.05	2.85	0.02	0.46
**Gender (χ^2^)**	**11.8 ***					-	**28.42 *****	0.51	3.33	2.98
Women	**30.2 (13) ^a^**	0 (0)	7.7 (1)	0 (0)	50 (1)	-	**87.5 (7) ^a^**	19.1 (13)	13 (7)	13.3 (8)
Men	**69.8 (30) ^b^**	100 (12)	92.3 (12)	100 (12)	50 (1)	-	**12.5 (1) ^b^**	80.9 (55)	87 (47)	86.7 (52)
**Marital Status (χ^2^)**	7.79					-	0.73	**6.12 ***	1.49	0.82
Married	74.4 (32)	83.3 (10)	84.6 (11)	83.3 (10)	0 (0) ^b^	-	62.5 (5)	**72.1 (49) ^b^**	72.2 (39)	73.3 (44)
Not Married	25.6 (11)	16.7 (2)	15.4 (2)	16.7 (2)	100 (2) ^a^	-	37.5 (3)	**27.9 (19) ^a^**	27.8 (15)	26.7 (16)
**Race and ethnicity (χ^2^)**	6.91					-	7.32	0.81	2.55	2.82
African American	28.6 (12)	33.3 (4)	41.7 (5)	18.2 (2)	0 (0)	-	50 (4)	30.3 (20)	26.4 (14)	25.9 (15)
Hispanic	26.2 (11)	16.7 (2)	25 (3)	36.4 (4)	0 (0)	-	50 (4)	24.2 (16)	22.6 (12)	24.1 (14)
Non-Hispanic White	40.5 (17)	50 (6)	33.3 (4)	45.5 (5)	100 (2)	-	0 (0) ^b^	42.4 (28)	49.1 (26)	48.3 (28)
Other	4.8 (2)	0 (0)	0 (0)	0 (0)	0 (0)	-	0 (0)	3 (2)	1.9 (1)	1.7 (1)
**Education (χ^2^)**	4.14					-	0.23	0.11	0.71	0.04
≤ Some college	39.5 (17)	33.3 (4)	53.8 (7)	50 (6)	100 (2)	-	37.5 (3)	45.6 (31)	48.1 (26)	45 (27)
≥ Associates degree	60.5 (26)	66.7 (8)	46.2 (8)	50 (6)	0 (0)	-	62.5 (5)	54.5 (37)	51.9 (28)	55 (33)
**Status (χ^2^)**	5.39					-	1.1	1.18	0.02	0.24
Active duty	69.8 (30)	58.3 (7)	84.6 (11)	91.7 (11)	100 (2)	-	87.5 (7)	69.1 (47)	72.2 (39)	70 (42)
Veteran	30.2 (13)	41.7 (5)	15.4 (2)	8.3 (1)	0 (0)	-	12.5 (1)	30.9 (21)	27.8 (15)	30 (18)
**Branch (χ^2^)**	2.65					-	0.18	0.17	1.03	0.06
Army	74.4 (32)	91.7 (11)	84.6 (11)	83.3 (10)	100 (2)	-	75 (6)	80.9 (55)	83.3 (45)	80 (48)
Other	25.6 (11)	8.3 (1)	15.4 (2)	16.7 (2)	0 (0)	-	25 (2)	19.1 (13)	16.7 (9)	20 (12)
**Time in Military (*F*)**	1.01						0.01	2.1	0.07	2.04
**Occupational Specialty (χ^2^)**	9.23					-	**8.02 ***	1.7	2.85	3.12
Combat arms	47.6 (20)	33.3 (4)	30.8 (4)	50 (6)	0 (0)	-	12.5 (1)	46.3 (31)	41.9 (26)	49.2 (29)
Combat support	23.8 (10)	33.3 (4)	15.4 (2)	33.3 (4)	0 (0)	-	12.5 (1)	23.9 (16)	26.4 (14)	25.4 (15)
Combat service support	28.6 (12)	33.3 (4)	53.8 (7)	16.7 (2)	100 (2) ^a^	-	**75 (6) ^a^**	29.9 (20)	24.5 (13)	25.4 (15)
**Rank (χ^2^)**	3.14					-	1.63	0.03	0.9	1.72
Enlisted	83.7 (36)	83.3 (10)	100 (13)	91.7 (11)	100 (2)	-	75 (6)	89.7 (61)	90.7 (49)	91.7 (55)
Officer	16.3 (7)	16.7 (2)	0 (0)	8.3 (1)	0 (0)	-	25 (2)	10.3 (7)	9.3 (5)	8.3 (5)
**Deployments, No. (χ^2^)**	**22.07 ***					-	4.94	**11.85 ****	**14.02 ****	**15.48 *****
1	19 (8)	33.3 (4)	0 (0)	25 (3)	0 (0)	-	12.5 (1)	**11.9 (8) ^b^**	**9.4 (5) ^b^**	**10.2 (6) ^b^**
2	40.5 (17)	33.3 (4)	23.1 (3)	16.7 (2)	50 (1)	-	62.5 (5)	37.3 (25)	35.8 (19)	35.6 (21)
3	26.2 (11)	**0 (0) ^b^**	**53.8 (7) ^a^**	8.3 (1)	50 (1)	-	0 (0)	28.4 (19)	**34 (18) ^a^**	**32.2 (19) ^a^**
≥4	14.3 (6)	33.3 (4)	23.1 (3)	**50 (6) ^a^**	0 (0)	-	25 (2)	22.4 (15)	20.8 (11)	22 (13)

Due to missing data, some categories may not represent 100% of the total sample of reported traumatic experiences. Significant findings presented in bold. * < 0.05, ** *p* < 0.01, *** *p* < 0.001. ^a^ indicates an adjusted residual (*z*) greater than 1.96. ^b^ indicates an adjusted residual (*z*) less than −1.96.

## References

[B1-behavsci-15-01211] American Psychiatric Association (2022). Diagnostic and statistical manual of mental disorders.

[B2-behavsci-15-01211] Chaumba J., Bride B. E. (2010). Trauma experiences and posttraumatic stress disorder among women in the United States military. Social Work in Mental Health.

[B3-behavsci-15-01211] Dardis C. M., Reinhardt K. M., Foynes M. M., Medoff N. E., Street A. E. (2018). “Who are you going to tell? who’s going to believe you?”: Women’s experiences disclosing military sexual trauma. Psychology of Women Quarterly.

[B4-behavsci-15-01211] Dedert E. A., Green K. T., Calhoun P. S., Yoash-Gantz R., Taber K. H., Mumford M. M., Tupler L. A., Morey R. A., Marx C. E., Weiner R. D., Beckham J. C. (2009). Association of trauma exposure with psychiatric morbidity in military veterans who have served since September 11, 2001. Journal of Psychiatric Research.

[B5-behavsci-15-01211] De Rond M., Lok J. (2016). Some things can never be unseen: The role of context in psychological injury at war. The Academy of Management Journal.

[B6-behavsci-15-01211] Griffin B. J., Purcell N., Burkman K., Litz B. T., Bryan C. J., Schmitz M., Villierme C., Walsh J., Maguen S. (2019). Moral injury: An integrative review. Journal of Traumatic Stress.

[B7-behavsci-15-01211] Guina J., Nahhas R. W., Sutton P., Farnsworth S. (2018). The influence of trauma type and timing on PTSD symptoms. Journal of Nervous & Mental Disease.

[B8-behavsci-15-01211] Hale W. J., Moore B. A., Straud C. L., Baker M. T., Peterson A. L. (2021). Examination of the factor structure and correlates of the perceived military healthcare stressors scale. Journal of Traumatic Stress.

[B9-behavsci-15-01211] Jakob J. M. D., Lamp K., Rauch S. A. M., Smith E. R., Buchholz K. R. (2017). The impact of trauma type or number of traumatic events on PTSD diagnosis and symptom severity in treatment seeking veterans. The Journal of Nervous and Mental Disease.

[B10-behavsci-15-01211] Jellestad L., Vital N. A., Malamud J., Taeymans J., Mueller-Pfeiffer C. (2021). Functional impairment in posttraumatic stress disorder: A systematic review and meta-analysis. Journal of Psychiatric Research.

[B11-behavsci-15-01211] Judkins J. L., Moore B. A., Collette T. L., Hale W. J., Peterson A. L., Morissette S. B. (2020). Incidence rates of posttraumatic stress disorder over a 17-year period in active duty military service members. Journal of Traumatic Stress.

[B12-behavsci-15-01211] Kilpatrick D. G., Resnick H. S., Milanak M. E., Miller M. W., Keyes K. M., Friedman M. J. (2013). National estimates of exposure to traumatic events and PTSD prevalence using DSM-IV and DSM-5 criteria: DSM-5 PTSD prevalence. Journal of Traumatic Stress.

[B13-behavsci-15-01211] Koenen K. C., Ratanatharathorn A., Ng L., McLaughlin K. A., Bromet E. J., Stein D. J., Karam E. G., Meron Ruscio A., Benjet C., Scott K., Atwoli L., Petukhova M., Lim C. C. W., Aguilar-Gaxiola S., Al-Hamzawi A., Alonso J., Bunting B., Ciutan M., De Girolamo G., Kessler R. C. (2017). Posttraumatic stress disorder in the world mental health surveys. Psychological Medicine.

[B14-behavsci-15-01211] Le Q. A., Doctor J. N., Zoellner L. A., Feeny N. C. (2018). Effects of treatment, choice, and preference on health-related quality-of-life outcomes in patients with posttraumatic stress disorder (PTSD). Quality of Life Research.

[B15-behavsci-15-01211] Macia K. S., Raines A. M., Maieritsch K. P., Franklin C. L. (2020). PTSD networks of veterans with combat versus non-combat types of index trauma. Journal of Affective Disorders.

[B16-behavsci-15-01211] Martin L., Rosen L. N., Durand D. B., Knudson K. H., Stretch R. H. (2000). Psychological and physical health effects of sexual assaults and nonsexual traumas among male and female united states army soldiers. Behavioral Medicine.

[B17-behavsci-15-01211] Norman S. B., Haller M., Kim H. M., Allard C. B., Porter K. E., Stein M. B., Venners M. R., Authier C. C., Rauch S. A. M. (2018). Trauma related guilt cognitions partially mediate the relationship between PTSD symptom severity and functioning among returning combat veterans. Journal of Psychiatric Research.

[B18-behavsci-15-01211] Peterson A. L., Blount T. H., Foa E. B., Brown L. A., McLean C. P., Mintz J., Schobitz R. P., DeBeer B. R., Mignogna J., Fina B. A., Evans W. R., Synett S., Hall-Clark B. N., Rentz T. O., Schrader C., Yarvis J. S., Dondanville K. A., Hansen H., Jacoby V. M., Keane T. M. (2023). Massed vs. intensive outpatient prolonged exposure for combat-related posttraumatic stress disorder: A randomized clinical trial. JAMA Network Open.

[B19-behavsci-15-01211] Peterson A. L., Foa E. B., Blount T. H., McLean C. P., Shah D. V., Young-McCaughan S., Litz B. T., Schobitz R. P., Castillo D. T., Rentz T. O., Yarvis J. S., Dondanville K. A., Fina B. A., Hall-Clark B. N., Brown L. A., DeBeer B. R., Jacoby V. M., Hancock A. K., Williamson D. E., Keane T. M. (2018). Intensive prolonged exposure therapy for combat-related posttraumatic stress disorder: Design and methodology of a randomized clinical trial. Contemporary Clinical Trials.

[B20-behavsci-15-01211] Stein N. R., Mills M. A., Arditte K., Mendoza C., Borah A. M., Resick P. A., Litz B. T., STRONG STAR Consortium, Belinfante K., Borah E. V., Cooney J. A., Foa E. B., Hembree E. A., Kippie A., Lester K., Malach S. L., McClure J., Peterson A. L., Vargas V., Wright E. (2012). A scheme for categorizing traumatic military events. Behavior Modification.

[B21-behavsci-15-01211] Steinert C., Hofmann M., Leichsenring F., Kruse J. (2015). The course of PTSD in naturalistic long-term studies: High variability of outcomes. A systematic review. Nordic Journal of Psychiatry.

[B22-behavsci-15-01211] Sun S., Pan W., Wang L. L. (2010). A comprehensive review of effect size reporting and interpreting practices in academic journals in education and psychology. Journal of Educational Psychology.

[B23-behavsci-15-01211] Vermetten E., Jetly R. (2018). A critical outlook on combat-related PTSD: Review and case reports of guilt and shame as drivers for moral injury. Military Behavioral Health.

[B24-behavsci-15-01211] Weathers F. W., Bovin M. J., Lee D. J., Sloan D. M., Schnurr P. P., Kaloupek D. G., Keane T. M., Marx B. P. (2018). The clinician-administered PTSD scale for DSM–5 (CAPS-5): Development and initial psychometric evaluation in military veterans. Psychological Assessment.

